# Cubital Tunnel Syndrome in Patients with and without Prior Elbow Trauma: A Retrospective, Observational Case Series

**DOI:** 10.1055/a-2977-6833

**Published:** 2026-10-15

**Authors:** Aurora A. Muça, Florian E. Dashi, Mishel G. Qirinxhi, Elizana Y. Petrela Zaimi, Arba F. Cecia, Ridvan H. Alimehmeti

**Affiliations:** 1 Service of Statistics ServiceUniversity of Medicine of Tirana, University Hospital Centre “Mother Theresa”TiranaAlbania; 2 Department of Neuroscience and NeurosurgeryNeurosciences Pavilion, University of Medicine of Tirana, University Hospital Centre “Mother Theresa”TiranaAlbania; 3 Department of NeurologyNeurosciences Pavilion, University of Medicine of Tirana, University Hospital Centre “Mother Theresa”TiranaAlbania; 4 Service of Anesthesiology and ReanimationUniversity of Medicine of Tirana, University Hospital Centre “Mother Theresa”TiranaAlbania; 5 Stritch School of Medicine, Loyola University ChicagoMaywoodIllinoisUnited States

**Keywords:** cubital tunnel syndrome, trauma, compression, fibrosis, deficits, McGowan

## Abstract

Cubital tunnel syndrome (CuTS) is the second most common peripheral nerve disorder of the upper limb and results from compression at anatomically vulnerable points along the course of the ulnar nerve. A total of 26 consecutive patients with CuTS who underwent surgical treatment by a single surgeon between January 2017 and March 2022 were retrospectively reviewed. Demographic characteristics, history of prior elbow trauma, clinical presentation, electrophysiological findings, surgical findings, and postoperative outcomes were analyzed. Three patients had documented major prior elbow trauma and were described individually. In the posttraumatic cases, preoperative neurological deficits were heterogeneous, and intraoperative findings included perineural fibrosis, altered tissue consistency, and an anomalous anconeus muscle. One of the three patients with prior elbow trauma improved by one McGowan grade, while two remained in the same grade at 12 months. All three demonstrated improvement in sensory symptoms, while motor recovery was variable and more limited in the patients with persistent higher-grade deficits. Postoperative complaints were limited to operative-site pain and transient paresthesia in two patients. Given the small number of posttraumatic cases, these findings are descriptive and hypothesis-generating rather than suitable for inferential comparison.

## Introduction

Cubital tunnel syndrome (CuTS) is the second most common entrapment neuropathy of the upper limb and results from compression of the ulnar nerve at anatomically vulnerable sites around the elbow. It is characterized by paresthesia, numbness, tingling, and pain along the ulnar nerve distribution, frequently accompanied by progressive motor weakness in advanced stages. CuTS remains underdiagnosed, potentially leading to delayed and irreversible neurological deficits. 1 2 3 4

The ulnar nerve originates from the C7–T1 fibers of the brachial plexus and courses posterior to the medial epicondyle before entering the cubital tunnel. This anatomical canal is bounded by the medial epicondyle, olecranon, Osborne ligament, and aponeurosis of the flexor carpi ulnaris (FCU). Along its course, the ulnar nerve provides motor innervation to several forearm and intrinsic hand muscles and sensory innervation to the ulnar aspect of the hand. 2 4 5 6 7

Several anatomical structures may contribute to ulnar nerve compression, including the cubital tunnel itself, the deep flexor-pronator aponeurosis, Struthers' arcade, Osborne ligament, and accessory muscles such as the anconeus epitrochlearis, reported in approximately 3 to 23% of individuals. CuTS occurs more frequently in men and has an estimated prevalence of approximately 6%, with a reported incidence of 20.9 cases per 100,000 person-years in the general population. The incidence may increase substantially in individuals exposed to occupational vibration or repetitive elbow strain, reaching up to 42.5% in certain worker populations. 1 3 8

The etiology of CuTS is multifactorial and may involve traumatic or non-traumatic mechanisms. Traumatic mechanisms include single-event injuries, such as fractures, dislocations, and significant blunt or sports-related trauma, whereas non-traumatic mechanisms include chronic external compression, repetitive elbow strain, and occupational exposure. 9 10 11 12 Prolonged nerve compression ultimately results in segmental demyelination, axonal degeneration, and perineural fibrosis, leading to progressive motor and sensory dysfunction. 2 5 8 11 13 14

CuTS clinical course is highly heterogeneous and influenced by the mechanisms contributing to ulnar nerve compression. Prior elbow trauma may induce structural alterations within the cubital tunnel—including fibrosis, scar formation, posttraumatic deformity, or altered joint biomechanics—that increase the vulnerability of the ulnar nerve to chronic mechanical stress. Despite trauma being recognized as a potential contributing factor, the current literature rarely distinguishes traumatic from non-traumatic CuTS with regard to disease progression, timing of diagnosis, or surgical outcomes. This creates a gap in understanding how prior elbow trauma shapes nerve vulnerability, chronicity of compression, and eventual surgical outcomes. Current CuTS management guidelines focus on anatomical compression sites and surgical technique, but do not address whether trauma-associated cases behave differently or require modified intervention strategies. Therefore, describing these characteristics may help generate hypotheses regarding the potential influence of prior trauma on the clinical course and surgical management of CuTS. 13

Our retrospective, observational study aimed to evaluate the clinical characteristics, surgical findings, and postoperative outcomes of patients with CuTS with and without prior elbow trauma, with particular emphasis on disease severity at presentation and neurological recovery following surgical decompression. 5

## Objective

This study aimed to describe the clinical characteristics, surgical findings, and postoperative outcomes of patients with CuTS with and without documented prior elbow trauma, with particular focus on disease severity at presentation and neurological recovery following surgical decompression.

## Materials and Methods

### Study Design and Patient Population

This retrospective, observational clinical study included 26 consecutive adult patients (≥ 18 years) diagnosed with CuTS who underwent surgical treatment at the Neurosciences Pavilion, Department of Neurosurgery, “Mother Theresa” University Hospital Center, between January 2017 and March 2022. All procedures were performed by a single neurosurgeon certified in peripheral nerve surgery, thereby constituting a single-surgeon retrospective study.

Patients were eligible for inclusion if they had clinical symptoms consistent with ulnar nerve entrapment at the elbow, electrophysiological confirmation by electroneurography (ENG), and an indication for surgical decompression. Patients were subsequently classified according to the presence or absence of documented major prior elbow trauma. Patients were excluded if they had valgus deformity of the elbow, space-occupying lesions such as tumors or ganglion cysts causing nerve compression, elbow instability, or mild/intermittent symptoms deemed suitable for conservative management.

### Data Collection

Clinical data were retrospectively retrieved from institutional medical records. Collected variables included demographic characteristics (age and sex), occupational status, history of prior elbow trauma, duration of symptoms, comorbidities, and results of preoperative investigations, including ENG and magnetic resonance imaging (MRI). Additional data included the site of nerve compression, intraoperative findings, type of anesthesia administered, and postoperative complications.

Functional severity was assessed both preoperatively and postoperatively using the modified McGowan classification.

### Surgical Technique

All patients underwent an in situ decompression of the ulnar nerve, performed under either local or general anesthesia. Intraoperative findings were systematically documented, including the presence of perineural adhesions, nerve caliber, and discrete points of compression.

### Definition of Trauma

For the purposes of this study, traumatic CuTS was defined as a documented history of a major, single-event elbow injury, such as fracture, dislocation, or significant blunt trauma, occurring before the onset of ulnar neuropathic symptoms. Non-traumatic CuTS was defined as the absence of such documented major elbow trauma. Occupational repetitive strain and chronic external compression were recorded when present but were not classified as traumatic for group allocation.

### Follow-Up

Patients were followed postoperatively at 1, 3, 6, and 12 months. McGowan grade was assessed at each follow-up visit. For all patients, the McGowan grade was recorded at each follow-up during the 12 months after intervention. All patients completed the 12-month follow-up.

### Statistical Analysis

Statistical analysis was performed using SPSS version 25.0 (Statistical Package for the Social Sciences, SPSS Inc.). Continuous variables were summarized using mean ± standard deviation or median and interquartile range, as appropriate, and categorical variables were presented as frequencies and percentages. Given the small number of patients with documented prior elbow trauma ( *n* = 3), analyses involving trauma status were considered descriptive. Preoperative and postoperative McGowan grades were summarized descriptively using patient-level transition data.

### Ethical Considerations

This study was conducted in accordance with institutional ethical standards for retrospective clinical research. All patient data were anonymized prior to analysis.

## Results

A total of 26 patients were included in the analysis, of whom 23 (88.5%) were classified as non-traumatic and 3 (11.5%) as traumatic cases. Regarding sex distribution, males comprised 2 of the 3 patients in the trauma group, whereas the non-trauma group included 17 males and 6 females. Given the small number of trauma cases, these distributions are presented descriptively.

Preoperatively, 11 patients in the overall retrospective study were classified as McGowan grade II and 15 as grade III. Among the three posttraumatic cases, two were classified as grade II and one as grade III. Given the very small number of posttraumatic cases, these findings are presented descriptively.

Two of the three patients in the traumatic group reported postoperative symptoms, consisting of pain at the operative site and transient paresthesia. These symptoms were self-limited and were not associated with neurological deterioration or reoperation. Given the very small traumatic subgroup, the frequency of these events should be interpreted descriptively rather than as evidence of an increased complication risk. These findings should be interpreted with caution due to the small sample size, particularly in the traumatic group, which limits statistical power.

The distribution of patients according to the McGowan classification, stratified by trauma status, demonstrates differing postoperative outcomes between groups.

In the non-trauma group ( *n* = 23), a clear postoperative shift toward lower severity grades was observed. Notably, no patients remained in grade III following surgery, while 69.6% were classified as grade 0 or I postoperatively. These findings indicate a favorable clinical response to surgical intervention in patients without traumatic etiology.

In contrast, the trauma group ( *n* = 3) demonstrated more limited and heterogeneous outcomes. No patients achieved grade 0 postoperatively; one patient improved from McGowan grade II to grade I, one remained in grade II, and one remained in grade III. Thus, one-third of patients demonstrated improvement by at least one McGowan grade, while two-thirds remained in the same grade. However, interpretation is constrained by the very small sample size.

Before surgery, 11 patients were McGowan II, and 15 were McGowan III. At 12 months, 3 were grade 0, 13 grade I, 9 grade II, and 1 grade III. The postoperative distribution appeared more favorable in the non-trauma group; however, the very small number of patients with prior trauma precludes meaningful between-group inference ( Table 1 ).

**Table 1:** Distribution of patients according to the McGowan classification, stratified by trauma status

Trauma	McGowan preop	McGowan postop				Total
		0	I	II	III	
No	II	3 (13.0)	5 (21.7)	1 (4.3)	0 (0.0)	9 (39.1)
	III	5 (21.7)	2 (8.7)	7 (30.4)	0 (0.0)	14 (60.9)
	Total	8 (34.8)	7 (30.4)	8 (34.8)	0 (0.0)	23 (100.0)
Yes	II	0 (0.0)	1 (33.3)	1 (33.3)	0 (0.0)	2 (66.7)
	III	0 (0.0)	0 (0.0)	0 (0.0)	1 (33.3)	1 (33.3)
	Total	0 (0.0)	1 (33.3)	1 (33.3)	1 (33.3)	3 (100.0)

The distribution of patients according to the McGowan classification before and after surgical intervention was analyzed in a retrospective study of 26 patients. Preoperatively, 11 patients were classified as grade II and 15 patients as grade III, indicating that the majority presented with advanced neurological impairment at baseline ( Table 2 ).

**Table 2:** General characteristics of the study population

Variables		Frequency	Percentage (%)
Sex	Male	19	73.1
	Female	7	26.9
Site	Left	10	38.5
	Right	14	53.8
	Bilateral	2	7.7
McGowan preop	II	11	42.3
	III	15	57.7
Trauma	No	23	88.5
	Yes	3	11.5
Comorbidities	No	10	38.5
	Yes	16	61.5
Age (years)	52.65 ± 11.74, median = 52.00, IQR = 12.75, range: 32–75		

Abbreviation: IQR, interquartile range.

Note: Age was presented as mean ± standard deviation.

Postoperatively, a clear shift toward lower severity grades was observed: Eight patients were classified as grade 0, eight patients as grade I, nine patients as grade II, and only one patient remained in grade III. Among patients initially classified as grade II, 81.8% (9/11) improved to grade 0 or I. Overall, 23 of 26 patients (88.5%) demonstrated improvement in McGowan grade, while three remained unchanged. In the non-trauma group, 69.6% (16/23) achieved grade 0 or I postoperatively. Overall, 61.5% of patients achieved grade 0 or I following surgery, indicating a shift toward lower McGowan grade after decompression ( Table 3 ).

**Table 3:** Distribution of patients according to the McGowan classification before and after surgical intervention

McGowan preop	McGowan postop				Total
	0	I	II	III	
II	3	6	2	0	11
	11.5%	23.1%	7.7%	0.0%	42.3%
III	5	2	7	1	15
	19.2%	7.7%	26.9%	3.8%	57.7%
Total	8	8	9	1	26
	30.8%	30.8%	34.6%	3.8%	100.0%

### Case Illustrations

#### Case 1

A 58-year-old, right-hand-dominant male mechanical engineer, with a documented history of major elbow trauma sustained during childhood 55 years ago, presented with right-sided ulnar neuropathy at our department. The patient had a known history of smoking for more than 22 years. He complained of paresthesia for more than 15 years. ENG demonstrated marked reduction in compound muscle action potential (CMAP) amplitude, slowed conduction velocity across the cubital sulcus, and severe temporal dispersion, consistent with McGowan grade II CuTS with demyelination and secondary axonal loss.

The patient underwent simple in situ decompression of the ulnar nerve, with preservation of the major sensory branches. Postoperatively, he reported immediate resolution of tingling. During follow-up at 1, 3, 6, and 12 months, he remained McGowan grade II. He reported occasional nocturnal pain in the ulnar nerve distribution, which persisted intermittently until approximately the eighth postoperative month. No persistent sensory deficit or neurological deterioration was documented ( Fig. 1 ).

**Fig. 1:**
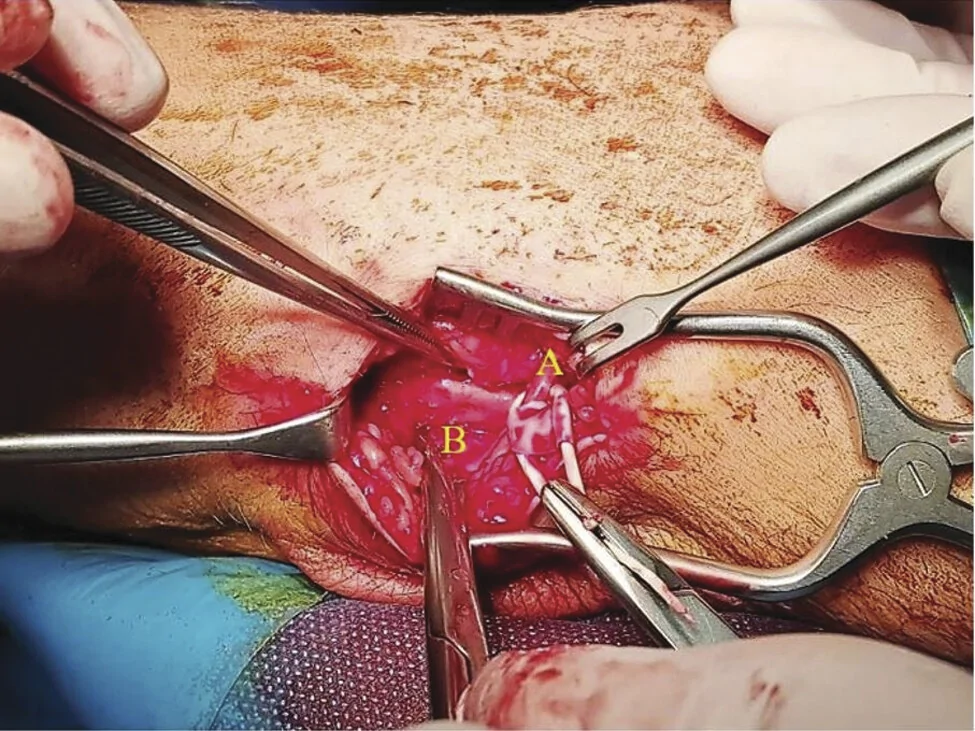
Intraoperative view of simple in situ decompression with evidence of compression sulcus track along the ulnar nerve (B) and sensory branch with considerable diameter preserved (A).

The patient sustained the elbow injury at approximately 3 years of age and subsequently experienced intermittent ulnar-distribution tingling and pain for several decades. The prolonged interval between the initial trauma and surgical treatment highlights the potential difficulty in recognizing chronic ulnar neuropathy when symptoms are intermittent and coexist with other elbow conditions, including ipsilateral olecranon bursitis.

#### Case 2

A 46-year-old male with a history of multiple prior elbow traumas, the most recent and important being 5 years earlier from a combustion injury, presented with progressive right elbow pain over the past year. The patient, employed at a gas station, also had a multilevel lumbar and thoracic degenerative disc disease documented on spinal computed tomography (CT). Clinical examination revealed deformation of the distal phalanges of the fourth digit, restricted extension of the right elbow, 90-degree limitation in elbow extension, an “ulnar claw” deformity of the right hand, and hypothenar hypotrophy. ENG confirmed right CuTS grade III McGowan with conduction block at FCU level with reduced CMAP amplitude and prolonged latency.

Intraoperatively, ulnar nerve compression was identified at the FCU fascial level, with increased adherence, fibrous consistency, and altered vascular configuration. Local joint sensory denervation was performed by dividing joint sensory branches, and selective division of cutaneous sensory branches supplying the peri-incisional skin was performed when necessary. Proximal sensory and arteriovenous branches at the level of Struthers' arcade were preserved.

Short-term follow-up demonstrated improvement in pain and partial recovery of sensory function on the right side. The patient was followed postoperatively at 1, 3, 6, and 12 months, complaining of occasional pain sensation. At each follow-up assessment, he remained grade III McGowan during all four follow-ups ( Fig. 2 ).

**Fig. 2:**
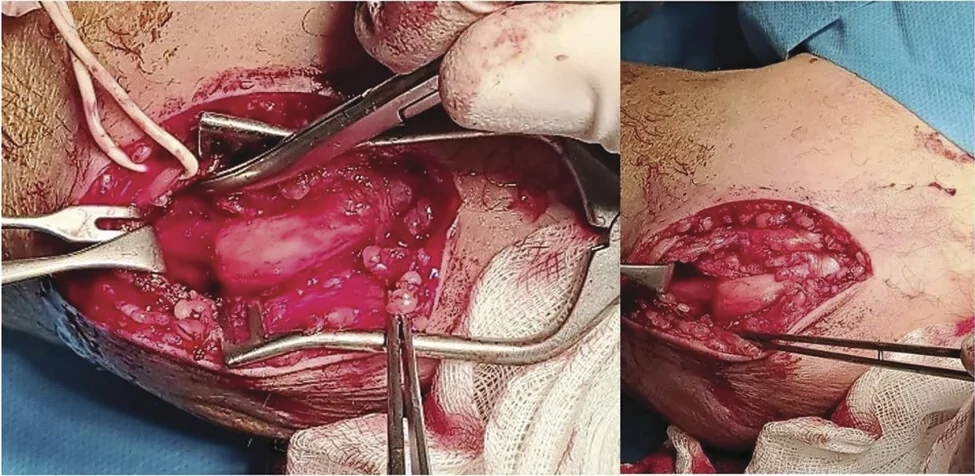
Evidence of a deep sulcus, caused by FCU fascia, over the ulnar nerve that has changed nerve and vascular conformation, as well as tissue consistency into fibrous tissue. Intraoperative view after simple in situ decompression was performed. FCU, flexor carpi ulnaris.

#### Case 3

A 39-year-old, right-hand-dominant female patient with a history of elbow trauma (documented fall without dislocation) 7 years earlier presented to our clinic with evidence of early-stage intrinsic hand muscle atrophy. Preoperatively, she was classified as McGowan grade IIB, with slight atrophy of the intrinsic hand muscles. Simple in situ decompression was performed. Intraoperatively, the presence of an anconeus muscle, a rare anatomical finding, was identified. The anomalous muscle was dissected to decompress the ulnar nerve. During the 1-year follow-up, the patient reported improved handgrip strength, slight recovery of the intrinsic hand muscles, and increased strength of the first finger flexion. At 1, 3, 6, and 12 months postoperatively, she reported no pain or sensory disturbances. Her McGowan grade improved from grade IIB preoperatively to grade I after the 6-month follow-up assessment and remained grade I at the 12-month follow-up.

## Discussion

Prior elbow trauma may be associated with differences in the clinical presentation and intraoperative findings of CuTS; however, the present study is descriptive and cannot establish a causal relationship. Traumatic events—including fractures, contusions, and iatrogenic injuries—may induce structural and vascular alterations of the ulnar nerve and surrounding tissue, thereby increasing susceptibility to chronic compression and neuropathic sequelae. All three patients with documented prior elbow trauma presented with McGowan grade II or III disease, indicating clinically significant neurological impairment at presentation. This observation highlights the potential impact of trauma not only on the timing of clinical presentation but also on the degree of preoperative nerve compromise, underscoring an important gap in current understanding of posttraumatic CuTS progression. 15 16

Notably, only three patients in our retrospective observational study explicitly reported a history of major elbow trauma. The remaining 23 patients (88.5%) had no documented major prior elbow trauma and were classified as non-traumatic according to the predefined study criteria. Cumulative minor injuries, including unrecognized contusions, repetitive elbow pressure, and occupational microtrauma, have been proposed as potential contributors to chronic nerve irritation and structural changes.

Our study findings are consistent with previously reported surgical outcomes, including a 2020 meta-analysis of 30 studies (2,894 operated limbs) reporting approximately 87% overall clinical improvement after decompression surgery.

## Pathophysiology

From a mechanistic perspective, ulnar neuritis primarily results from compression within the cubital tunnel, a process exacerbated during elbow flexion. Flexion generates tractional forces that increase intraneural tension, impede axoplasmic flow, and compress the vascular supply of the nerve, ultimately producing ischemia and intraneural edema. Repetitive or sustained compression promotes progressive fibrosis, thickening of connective tissue sheaths, and impaired gliding of the nerve both intra- and extraneuronally. These alterations disrupt the physiological microenvironment of the nerve, impair retrograde and anterograde axonal transport, and reduce the delivery of neurotrophic factors to the neuronal cell body. Clinically, such pathophysiological changes manifest as progressive paresthesia, numbness, and motor weakness, corresponding to increasing McGowan grades. 1 3 10 11 17 18 19 20

The role of aberrant anatomical structures, such as the anconeus epitrochlearis muscle, may further complicate posttraumatic CuTS. While some studies suggest that this muscle may protect the ulnar nerve by increasing the available space within the cubital tunnel, hypertrophy or posttraumatic swelling may conversely make it a potential source of compression. Similarly, anatomical variations of the Osborne ligament may influence nerve displacement during elbow flexion, predisposing the nerve to chronic dynamic compression, particularly following trauma. These anatomical factors might be less clinically evident in non-traumatic CuTS, but might become significant in posttraumatic cases, where tissue remodeling and fibrosis further increase nerve vulnerability. 13 20 21 22

## Clinical Implications

Posttraumatic ulnar neuropathy may follow a clinical trajectory distinct from that of non-traumatic CuTS. Following elbow trauma, progressive structural remodeling—such as perineural fibrosis, scar formation, or subtle posttraumatic deformity—may increase mechanical constraints within the cubital tunnel and exacerbate chronic nerve compression. However, recognition of neuropathic symptoms may be delayed because early manifestations can be masked by trauma-related pain, edema, or temporary joint immobilization during the acute recovery phase. As a result, patients may present only after neurological deficits have progressed, often corresponding to higher McGowan grades and more pronounced motor and sensory impairment, as evidenced in this case series. 1

Electrophysiological studies often corroborate this severity, demonstrating reduced conduction velocities and prolonged distal latency along the ulnar nerve. Although electrodiagnostic testing remains operator-dependent, it provides objective localization and quantification of nerve dysfunction and contributes to surgical planning. Advanced imaging modalities, including MRI and high-resolution ultrasound, may further delineate structural abnormalities—such as nerve thickening, fibrosis, or anomalous musculature—thereby complementing clinical and electrophysiological evaluation. 7 11 20 23 24 25

## Surgical Considerations

Early surgical intervention, generally considered within 3 to 4 weeks following traumatic injury in the relevant literature, may favor neurological recovery by limiting the duration of nerve compression and secondary neural injury. However, all three posttraumatic patients in the present series underwent surgical treatment long after the initial traumatic event; therefore, this study cannot evaluate the effect of early versus delayed intervention. The prolonged interval between trauma and treatment may nevertheless be relevant when interpreting the persistent motor deficits observed in these cases.

Previous studies suggest that patients with trauma-associated CuTS can achieve postoperative outcomes comparable to those of non-traumatic cases when appropriate surgical techniques are employed, although preoperative deficits may be more pronounced. In particular, removal of hypertrophied or aberrant muscles has been shown to enhance grip and pinch strength postoperatively, emphasizing the importance of individualized surgical strategies based on intraoperative anatomical findings. 3 26 27 28 29 30 31 32 33

## Knowledge Gaps and Limitations

Despite favorable outcomes observed in our retrospective observational study, several knowledge gaps persist. The limited size of traumatic cohorts in the current literature—including the present study—restricts the ability to draw definitive conclusions regarding the impact of early versus delayed decompression on long-term functional recovery.

Similarly, the relationship between specific trauma mechanisms, aberrant anatomical structures, and postoperative outcomes has not been systematically characterized. Most available evidence derives from retrospective studies with heterogeneous reporting standards, which limits the development of standardized prognostic criteria.

Furthermore, the influence of repeated microtrauma or chronic posttraumatic changes on nerve regeneration and remyelination may modulate recovery speed and completeness, but their precise clinical relevance requires confirmation in larger prospective investigations. 13 34

## Prognosis

The postoperative complaints observed in two traumatic cases consisted of operative-site pain and transient paresthesia rather than major neurological or surgical complications. The paresthesia was also present preoperatively, although patients described its postoperative character differently during follow-up. These symptoms may reflect the postoperative course of a chronically compressed and structurally altered nerve undergoing recovery after decompression, but a causal relationship cannot be established from this small series.

Postoperative outcomes in traumatic CuTS may be favorable, although recovery can be variable and may depend on the severity and chronicity of nerve injury. The postoperative course of the three traumatic cases in the present series was not associated with major neurological or surgical complications, although persistent motor deficits were observed. Rehabilitation strategies—including controlled mobilization, splinting, and activity modification—are important to optimize nerve gliding and reduce the risk of recurrent compression. Failure to address posttraumatic anatomical changes may result in persistent deficits, underscoring the importance of comprehensive preoperative assessment and individualized surgical planning. 21 35 36

## Conclusion of Discussion

In conclusion, prior elbow trauma is suggested to influence the clinical presentation and surgical complexity of CuTS. The three posttraumatic cases presented with clinically significant neurological deficits and heterogeneous electrophysiological findings. Given the small number of cases, these observations are descriptive and cannot establish whether prior trauma is associated with greater preoperative severity.

The postoperative course of the three patients with prior elbow trauma deserves consideration beyond changes in McGowan grade. One of the three patients improved by one McGowan grade, from grade II to grade I, whereas the remaining two patients remained at their preoperative grades at 12 months. Nevertheless, all three patients demonstrated clinical improvement in sensory symptoms. The first patient reported immediate resolution of tingling, the second experienced improvement in pain and partial sensory recovery, and the third reported no residual pain or sensory disturbances during follow-up, together with improvement in handgrip strength and slight recovery of the intrinsic hand muscles. These findings suggest that symptomatic improvement may occur even when the overall McGowan grade remains unchanged. Thus, an unchanged McGowan grade did not necessarily indicate absence of clinical benefit, as two patients experienced symptomatic improvement despite remaining at the same McGowan grade, while the third patient demonstrated both symptomatic improvement and a one-grade improvement in McGowan classification.

This distinction may be particularly relevant in patients with longstanding motor deficits, in whom decompression can relieve the ongoing mechanical insult but may not immediately reverse established axonal injury and chronic structural changes. Prolonged nerve compression may result in demyelination, axonal loss, intraneural ischemic changes, and perineural fibrosis; consequently, sensory symptom relief may precede measurable motor recovery. Recovery of longstanding motor deficits may require a longer period and may remain incomplete despite adequate decompression. In patients who remained at the same McGowan grade at 12 months, this persistence may reflect the chronicity and severity of preoperative nerve injury rather than failure of decompression itself.

Given the limited sample size of this study, these findings should be interpreted cautiously and considered hypothesis-generating rather than definitive. Important knowledge gaps remain regarding the optimal timing of intervention, prognostic factors influencing neurological recovery, and the interaction between trauma-related anatomical changes and postoperative nerve regeneration. Future prospective studies with larger cohorts and standardized reporting are necessary to better define these relationships and guide clinical decision-making. 3 6 8 10 20 27 37 38 39 40 41

## Conclusion

In this retrospective case series, the three patients with prior elbow trauma demonstrated clinically heterogeneous presentations characterized by chronic sensory and motor deficits and, in some cases, substantial structural alterations around the ulnar nerve. Intraoperative findings included perineural fibrosis, altered tissue consistency, and an anomalous anconeus muscle, emphasizing the importance of careful intraoperative assessment and adequate decompression. At 12 months, one patient improved by one McGowan grade, whereas two remained in their preoperative grade. Nevertheless, all three patients demonstrated improvement in sensory symptoms, with variable motor recovery. This clinical discrepancy might indicate that sensory recovery could precede recovery of longstanding motor deficits following decompression. Postoperative complaints were limited to operative-site pain and transient paresthesia in two patients, without evidence of major neurological deterioration. Given the very small number of traumatic cases, these observations should be regarded as descriptive and hypothesis-generating. Larger studies are needed to clarify the influence of trauma chronicity, severity of preoperative nerve injury, and timing of decompression on long-term motor and sensory recovery.

## Highlights

- Prior elbow trauma may contribute to delayed presentation and advanced neurological deficits in CuTS.
- Clinical improvement may occur despite limited change in McGowan grade following decompression.
- Intraoperative findings in posttraumatic cases included perineural fibrosis and anatomical alterations around the nerve.
- Careful intraoperative assessment may help guide individualized management of trauma-associated ulnar neuropathy.

## Data Availability

The data supporting the findings of this study are available within the hospital archive. Anonymized data may be made available from the corresponding author upon reasonable request and with institutional permission.
